# Implementing Autonomous Control in the Digital-Twins-Based Internet of Robotic Things for Remote Patient Monitoring

**DOI:** 10.3390/s24175840

**Published:** 2024-09-09

**Authors:** Sangeen Khan, Sehat Ullah, Khalil Ullah, Sulaiman Almutairi, Sulaiman Aftan

**Affiliations:** 1 Department of CS and IT, University of Malakand, Chakdara 18800, Pakistan; sehatullah@uom.edu.pk; 2 Department of Software Engineering, University of Malakand, Chakdara 18800, Pakistan; khalil.ullah@uom.edu.pk; 3 Department of Health Informatics, College of Applied Medical Sciences, Qassim University, Buraydah 51452, Saudi Arabia; ssmtiery@qu.edu.sa; 4 Department of Computer Science, Texas Tech University, Lubbock, TX 79409, USA; sulaiman.aftan@ttu.edu

**Keywords:** autonomous robots, Internet of Things, Internet of Robotic Things, remote patient monitoring, digital twins

## Abstract

Conventional patient monitoring methods require skin-to-skin contact, continuous observation, and long working shifts, causing physical and mental stress for medical professionals. Remote patient monitoring (RPM) assists healthcare workers in monitoring patients distantly using various wearable sensors, reducing stress and infection risk. RPM can be enabled by using the Digital Twins (DTs)-based Internet of Robotic Things (IoRT) that merges robotics with the Internet of Things (IoT) and creates a virtual twin (VT) that acquires sensor data from the physical twin (PT) during operation to reflect its behavior. However, manual navigation of PT causes cognitive fatigue for the operator, affecting trust dynamics, satisfaction, and task performance. Also, operating manual systems requires proper training and long-term experience. This research implements autonomous control in the DTs-based IoRT to remotely monitor patients with chronic or contagious diseases. This work extends our previous paper that required the user to manually operate the PT using its VT to collect patient data for medical inspection. The proposed decision-making algorithm enables the PT to autonomously navigate towards the patient’s room, collect and transmit health data, and return to the base station while avoiding various obstacles. Rather than manually navigating, the medical personnel direct the PT to a specific target position using the Menu buttons. The medical staff can monitor the PT and the received sensor information in the pre-built virtual environment (VE). Based on the operator’s preference, manual control of the PT is also achievable. The experimental outcomes and comparative analysis verify the efficiency of the proposed system.

## 1. Introduction

In conventional patient monitoring methods, medical personnel keep manual records and continuously monitor patients’ health. Hospitals have limited resources, thus manually taking patients’ vital signs depends on many factors, including clinical workload, staff working hours, and patient diagnosis [1]. Furthermore, invasive devices are used for patient monitoring, which requires skin-to-skin contact to estimate vital signs [2]. This raises the possibility of exposing medical personnel to infectious diseases because of their asymptomatic nature and high transmission rate [3]. Healthcare practitioners not only undergo the common fear of being infected but also deal with other stresses, such as fear about the safety of their families, and the deaths of colleagues [4]. Remote patient monitoring (RPM) can complement conventional treatment and provide an alternative that benefits patients’ and care providers’ social and financial well-being [5]. RPM gathers and transmits patient health data to medical professionals using various digital health technologies [6]. The data acquired by RPM areassessed by healthcare practitioners to implement modifications in patients’ treatment procedures [7]. It is an essential tool for health carers to monitor and treat infected patients and those with chronic conditions [8]. It improves patient management and care capacities by enabling health professionals to spot diseases earlier and remotely examine chronic or contaminated patients, and those recovering from surgeries inside the hospitals or at home [2,9]. However, implementing RPM is a complicated and challenging task [10]. Many innovative technologies have been used to design and implement frameworks for RPM [11].

The Digital-Twins (DTs)-based Internet of Robotic Things (IoRT) [12] is the best candidate for RPM. The DTs-based IoRT integrates the Internet of Things (IoT) and robotics and creates a virtual replica [virtual twin (VT)] of the physical robotic thing [physical twin (PT)] that receives real-time data from the PT to update the status of VT in the virtual environment (VE) [13]. It combines virtual and physical spaces, allowing synchronized operation of the physical and virtual entities [14]. When the physical twin (PT) is altered, the virtual twin (VT) is updated automatically to reflect the same changes [15]. This framework consists of three main components, i.e., physical object, virtual replica, and bi-directional data connection between them. Integrating the physical and virtual entities enables the virtual model to accurately mimic the physical system, allowing the physical object to adjust its status using the feedback from the virtual entity when needed [16,17].Monitoring patients remotely through robotic systems is essential to medical care [18]. It saves significant time and notifies physicians earlier during the emergency to save the patient’s life [19]. Robotic things (RTs) can autonomously act or react to changes occurring in their surroundings [13]. They can potentially combat infectious diseases because they resist microbes and can arrive at places independently where human access is nearly impossible or dangerous [20,21]. They can be used in hospitals for disinfecting surfaces [22], mopping and cleaning [23], and drug distribution [24]. Combining robots with IoT devices and sensors provides real-time information regarding patient health and reduces the risk of human errors in prescribing medication and procedures [25]. It enhances the capacity of IoT through active sensing and actuation via robotic devices/things [26].

However, manually navigating the robotic things is a time-consuming and complex task. It causes extra cognitive fatigue for the operator because of increased task complexity or demand for additional situational awareness, affecting trust dynamics, complacency, and job performance. Also, operating robotic systems requires proper training and long-time experience.

Autonomous robotic systems can efficiently deal with these issues. They can enable the treatment and monitoring of patients with chronic and infectious diseases, replacing or distributing the responsibilities of the healthcare workers performing complex tasks, and improving the overall medical care services [27]. Also, they can traverse a path towards their destination without encountering obstructions in the physical environment [28], eliminating the workload and fatigue experienced by medical staff during manual navigation.

This paper presents a real-time RPM system that implements autonomous control in the DTs-based IoRT. The proposed work is an extension of our previous published study [12], in which the medical staff had to manually navigate the PT to the destination by operating its VT to collect health data from the biomedical sensors of the patient. A pre-built virtual environment (VE) was used to provide an accurate perception of the PT’s surroundings and visualize the collected health data for medical inspection.

The proposed system enables the PT to arrive at the patient room autonomously, collect health-related data from the patient-mounted sensors, transmit the information to the base station for medical examination, and return to the medical service. The developed decision-making algorithm can efficiently navigate the PT while evading various obstacles. Rather than manually navigating the PT to the target, the operator only has to initiate it towards a specific destination (patient room) by selecting one of the buttons (Room 1, Room 2, Room 3, Room 4) from the Menu. The healthcare workers can observe the PT and the health information of the infected patient in the VE. Switching from autonomous to manual mode is achievable depending on the user’s preference.

### 1.1. Motivation

Currently, due to rising medical complications, population growth, and various pandemics, robotic-based RPM has become increasingly important. Monitoring patients remotely using robotic devices saves significant time and notifies physicians earlier during the emergency to save the patient’s life. They can combat infectious diseases since they are immune to microbes and can travel to areas where human access is either difficult or dangerous. However, the manual operation of robotic systems causes cognitive fatigue for the operators because of increased task complexity or demand for additional situational awareness, affecting trust dynamics, complacency, and job performance [29]. Also, operating the robotic systems requires proper training and long-term experience [30]. This study aims to safeguard the robot operators from the metal fatigue they encounter while navigating robotic devices manually. Enabling the RTs to navigate autonomously for RPM can resolve these issues.

### 1.2. Contribution

This research implements autonomous control in the DTs-based IoRT to remotely monitor patients with chronic or infectious diseases. The main contributions are:

- Our proposed system enables the operator to remotely monitor the PT’s autonomous navigation in the VE and switch to manual control when needed.
- We developed and implemented a decision-making algorithm for autonomous navigation and an obstacle avoidance mechanism that uses various geometrical patterns to evade obstructions for path recalculation.
- We analyzed the performance of PT’s navigation and patient monitoring setup and performed a comparative analysis of the RPM systems and virtual reality (VR) and DTs frameworks.

The remaining part of thispaper is structured as follows: Section 2 outlines the related work, Section 3 discusses the proposed work, Section 4 describes the experimental setup and performance evaluation, Section 5 presents the discussion, and Section 6 presents the conclusion and future work.

## 2. Related Work

This section provides a summary of the research studies relevant to our topic. In this era of pandemics and increased medical complications, robot-based RPM is an essential tool for healthcare practitioners to treat infected or chronic patients. In this regard, we reviewed the research publications that used robotic devices for RPM. The RPM systems described in the following section are based on robots that can be operated manually or autonomously.

Khan et al. [31] developed a manually navigating mobile Robot Doctor (RoboDoc) that measures vital signs to remotely monitor the health of COVID-19 patients without directly interacting with them. A desktop-based GUI is developed to visualize vital signs for monitoring. A tablet with a mobile application is used to enable conversation between patients and medical staff. The robot includes a DSLR camera connected to a Raspberry Pi 4 that communicates with the server via Wi-Fi.

Mamun et al. [32] updated their iWard robot for remotely monitoring hospitalized patients’ health conditions. The robot collects and analyzes the vital signs to monitor the patient’s physical condition. The health information is then transmitted to a server for storage and examination by healthcare professionals. Each patient is assigned a unique identity to differentiate between their records.

Mišeikis et al. [33] adjusted their autonomous assisting robot (Lio) to perform additional functionalities during the COVID-19 pandemic. Lio can monitor the body temperature of passers-by and perform disinfection of surfaces using UV-C light. The system uses a thermal camera to measure the body temperature remotely. In case of elevated temperature, the medical staff is notified to collect manual data of the suspected persons using a standard medicinal thermometer. Lio has a powerful onboard computing unit; thus, data processing is performed without transmitting the data to cloud services.

Cantone et al. [34] presented an IoRT system to remotely monitor the health conditions of elderly people, transmitting their vital signs to medical assistants and physicians to ensure appropriate care. Conversation between elderly people, physicians, and care providers is allowed through an external Telegram boat. The communication is provided using Wi-Fi, IEEE 802.22, and Ethernet.

Rai et al. [35] designed and developed an autonomous virtual doctor robot (VDR) that can distantly monitor patients with COVID-19 without any physical contact. The VDR collects the patient’s vital signs using various sensors and transmits them to the medical practitioners via a Wi-Fi network. A mobile application (Blynk app) is developed that allows doctors to interact with the robot and receive health-related information over the Internet cloud.

Mireles et al. [36] proposed a nursing robot that can remotely monitor a patient’s vital signs (oxygen level, heart rate, blood pressure, and temperature). The robotic device can also assist people in the gait cycle. A graphical user interface (GUI) is used to present the data collected and processed by the system. Furthermore, the GUI is used for interaction between the robot, patients, and doctors. The ZigBee modules are used for communication between various entities of the system.

Some researchers proposed a RPM system based on a robotic device that can work in both autonomous and manual modes.

Antony et al. [37] developed a medicine delivery and RPM robot capable of navigating in autonomous and manual modes. An infrared sensor is attached to the bottom of the robot to identify the path, and an ultrasonic sensor is mounted to the front to detect the obstacles. The collected parameters (temperature, pulse rate) are sent to the doctor through the Internet for inspection. An android application is developed that communicates with the robot via Bluetooth device (HC-05) to receive the parameters.

Interaction and visualization are the key components for remote operations of robotic systems. We reviewed the related articles that used VR and DTs-based interfacing and supervision techniques to facilitate different aspects of life.

Some researchers leveraged the advantages of VR-based techniques to remotely operate a mobile robot.

Solanes et al. [38] developed a VR-based system for remotely controlling a mobile robot that accomplishes various tasks including, bomb disposal, and human rescue.The VR interface allows the user to have a better view of the physical scenario and real-time human–robot interaction. The human operator directs the robot through the environment using its intellectual abilities, while the robot avoids collisions in space to take advantage of its quick response. The VE presents various elements needed for remote operation such as user reference, mobile robot, two-dimensional (2D) map of the environment, information regarding the task or robot, and the position of the real-time detected objects. A gamepad is used to interact with the VE for a longer time.

Many researchers integrated VR and DT approaches to monitor and control robots performing various tasks.

Topini et al. [39] designed a robotic hand exoskeleton for rehabilitation based on integrating VR and DTs. The predefined VE is developed using the Webot framework. Mapping between the DTs of the hand exoskeleton is performed to mimic the status of the physical twin in the VE and enable the patient to physically observe the virtual objects on hand via force feedback. As long as the virtual hand is not in contact with a barrier, the system remains in a free-motion state. When the virtual model encounters an obstruction, the virtual force values are sent to the physical hand exoskeletonas a reference signal for force feedback.

Kalinov et al. [40] developed a VR-based user interface for supervising an autonomous robot that transports stock in a warehouse to avoid the spread of COVID-19 between people. The virtual interface allows inexperienced warehouse workers to operate the heterogeneous robotic framework comprising an unmanned ground vehicle (UGV) and an unmanned aerial vehicle (UAV). It visualizes the virtual model of the system in the DT of the warehouse’s physical environment and a live view of real space from the onboard camera of the aerial vehicle. The user can interact with the interface via a hand-held VR device.

Ponomareva et al. [41] presented a robot manipulation system based on a VR interface. The region-based convolutional neural network detects the laboratory instruments and calculates their location in real space for visualization in the VE. The DT informs the operator about the position of the real robot. The VE represents the robot’s current status from different viewpoints without using complex camera-based techniques. The haptic device’s handle is used to direct the robot’s motion. At the end of the robot’s gripper, a visual camera is mounted to assist in the manipulation process.

Grag et al. [42] created a DT model of an industrial robot that provides synchronized control of the physical robot over a specified trajectory. This framework only supports FANUC robots and communicates over client/server architecture. A VE is created to exhibit the DT and provide natural interaction with the DT and the physical robot. A VR device is used to control both the virtual and physical robots’ movements.

Taheema et al. [43] developed a DT model that can be used to control the industrial manufacturing system in real time. A VE is created to display the robotic system’s DT. The system displays the robotic cells and production lines and controls the entire process.

Laaki et al. [44] created a DT of the robotic arm intended to perform remote surgery. The DT and the real robot are mapped for synchronized operation. The DT is displayed in a virtual environment (VE) designed to provide an immersive experience. The virtual space resembled a medical setup and included a virtual model of a dummy patient. The movement of the head-mounted display (HMD) and handheld controllers is tracked using an infrared laser grid.

According to the literature review, DTs-based robots and VR have been used in industrial manufacturing, inspection tasks, patient rehabilitation, remote monitoring of autonomous robots, and remote surgeries. However, none of the existing systems has its applications RPM. Also, they can only monitor or control a specific entity. They cannot connect to external sensors or devices for data acquisition or transmission. In robot monitoring, autonomous robot supervision has been suggested in only one paper [40]. However, the study lacks navigation performance evaluation and comparison with the existing studies.

Most existing works on robot-based RPM do not include evaluation protocols to measure the robot’s navigation accuracy. They lack measuring DQ dimensions for the monitoring data. Furthermore, they do not present a comparative analysis to verify the efficiency of the proposed frameworks over the existing systems.

The proposed system implements autonomous control in the DTs-based IoRT to remotely monitor patients with chronic or contagious diseases. The system enables monitoring of the autonomous robotic device in VE and allows its connections with external sensors and devices for RPM. Unlike the existing frameworks, the proposed approach includes an evaluation protocol to calculate navigation accuracy. It also measures the DQ dimensions of the monitoring data. It presents a comparison of the existing and the proposed approaches in navigation and DQ dimensions. It also provides a comparison of VR and DTs-based robotic systems. Furthermore, the framework allows manual control of the PT based on the user’s preference.

## 3. The Proposed System

**Figure 1:**
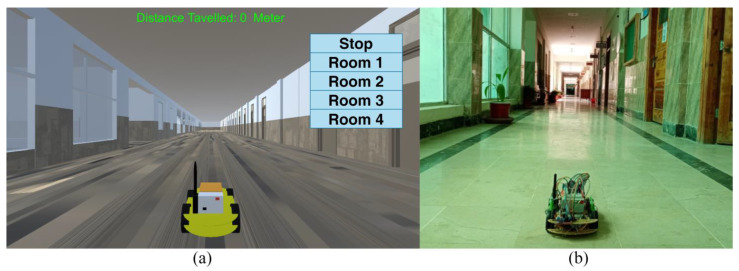
(**a**) VT in the VE. (**b**) PT in the RE.

This proposed system implements autonomous control in the DTs-based IoRT to remotely monitor patients with chronic or contagious diseases. The system’s components and operation are discussed in the following part. The framework comprises physical and virtual twins (DTs) connected through a communication link. The VT is visualized in the VE to depict the status of the PT in real time, as shown in Figure 1.

The data acquisition from the patient monitoring unit (PMU) is carried out using Bluetooth modules (HC-05). The radio transceivers (NRF24L01+) are employed for communication between the PT and the health service. A laptop PC is used for virtual renderings, data processing, and visualization.

**Figure 2:**
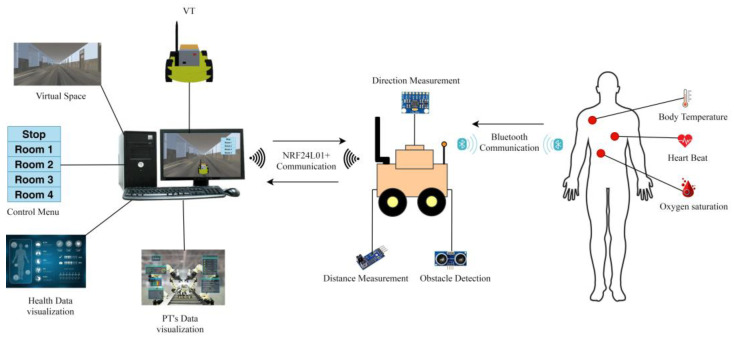
Graphical abstract of the proposed system.

The PT navigates autonomously in the RE for RPM. After arriving at the patient’s room, it connects with PMU and receives sensor information for 10 s. The health parameters are then transmitted to the medical service and displayed in the VE for medical inspection. The VT in the virtual space is used to monitor the PT operating in the actual environment. Because of the autonomous nature of the proposed system, medical staff is relieved from the manual controlling load and mental fatigue. The operator only has to monitor the VT in the virtual space to observe the PT’s actual status in the RE. Manual control of the PT depends on the operator’s preference. The proposed system diagram is shown in Figure 2, which includes all the main elements of the virtual setup, the physical robotic device, and the patient monitoring setup.

The key components that should be considered while designing any autonomous robotic system are locomotion, perception, cognition, and navigation, as described in [45,46]. The proposed system is built on the four important elements used for autonomous navigation.

### 3.1. Locomotion

The first step in building an autonomous robot is locomotion. Even though robots often move in safe and controlled areas, they must occasionally traverse in extreme or unknown settings. The robots may also function in other environments, such as air or water. Autonomous robots rely not merely on control but also on locomotion systems. Locomotion is an important subject for creating autonomous robotic systems, and it is dependent not only on the physical environment in which the robots navigate, but also on various technological factors such as maneuverability, stability, and efficiency. The proposed system leverages a four-wheeled mobile robotic thing (PT) that moves through the corridor autonomously, collecting health parameters from biomedical sensors and transmitting them to a medical service for examination. The PT moves inside the indoor space on a flat surface, hence a wheeled robot is preferable to a legged or treaded device. Wheeled robots do not pose balance issues because they are generally in contact with the ground surface.

### 3.2. Perception

Navigating autonomous mobile robots requires vital information regarding the surrounding environment and the robot itself. This is achieved through the robot’s onboard sensors, subsequently, relevant information is extracted from the sensors’ calculations. The sensor data areused to conduct the robot positioning, representation, and mapping tasks. Currently, a number of sensors are available that enable robotic devices to know about the surroundings and activities around them, such as RGBD Cameras, LiDARs, and Sonars. However, selecting one or many from these is wide, and based on the specific requirements [47]. The proposed system’s PT consists of various sensors to gather real-time information concerning the navigation and position of the PT. A speed sensor (LM393) is used whose values are utilized to calculate the distance covered by the PT in the RE. Three ultrasonic distance sensors (HC-SR04) are fitted with the robot for obstacle detection and avoidance, providing the user with situational awareness. The sensors are mounted on the left, right, and front sides of the PT. An accelerometer/gyroscope sensor (MPU6050) is installed to calculate the robot’s direction in the RE.

### 3.3. Cognition

Once the environment knowledge and the robot’s direction and destination are known, the cognitive system plans the path to achieve the objectives. Hence, the cognitive phase is referred to as the decision-making and implementation phase. Using the sensor information, the cognition system decides the next action to achieve the goal. In the context of a mobile robot, the exact aspects of the cognition phase are directly connected with the robot’s robust navigation. The following section explains the proposed system’s obstacle avoidance and path calculation criteria.

#### 3.3.1. Obstacle Avoidance

The ultrasonic distance sensors’ (HC-SR04) values are used to detect, visualize, and avoid both static and dynamic obstructions in real time. When the sensor detects an obstruction closer to the PT than the threshold distance (0.4 m), it is visualized as a cube to provide the user with situational awareness. The distance values are also displayed in the virtual space, along with their labels (Left, Right, and Front) to provide more information about the detected obstacle. If there are no obstacles, the PT has to travel the straight path towards the target position (TP) and then back to the health center. If the sensors detect obstructions within the threshold distance, the decision-making algorithm analyzes them to ensure safe navigation. The barriers to the left, right, or left and right are ignored by the PT as they do not affect its movement towards the TP. If the barrier is to the front side, the PT stops moving, turns towards the right (45°), and moves forward (0.5 m) to avoid the obstacle. After evading the barrier, it turns towards the left (90°) and moves forward (0.5 m). Finally, it takes a right turn (45°) to align with the straight path to reach the destination. Similarly, if the barriers are to the front and left sides, the PT stops moving, turns towards the right (45°), and moves forward (0.5 m) to avoid the obstacles. After evading the barriers, it turns towards the left (90°) and moves forward (0.5 m). Finally, it takes a right turn (45°) to align with the straight path to reach the destination. If the obstacles are to the front and right sides, the PT stops moving, turns towards the left (45°), and moves forward (0.5 m) to avoid the obstacles. After evading the obstructions, it turns towards the right (90°), and moves forward (0.5 m). Finally, it takes a left turn (45°) to align with the straight path to reach the desired TP. However, if the obstructions are to the front, left, and right sides, the PT stops moving, moves backward (0.5 m), turns towards the right (90°), and moves forward (0.7 m). Then, it turns left (90°) and moves forward (1 m) to avoid the obstacles. After evading the obstructions, it turns towards the right (45°) and moves forward (0.8 m). Finally, it takes a right turn (45°) to align with the straight path to reach the particular TP.

#### 3.3.2. Path Calculation

During obstacle avoidance, the PT covers an additional distance and stops before reaching the predefined destination. Therefore, the path is recalculated to enable accurate navigation. The proposed system uses various patterns and functions to recalculate the path.

**Figure 3:**
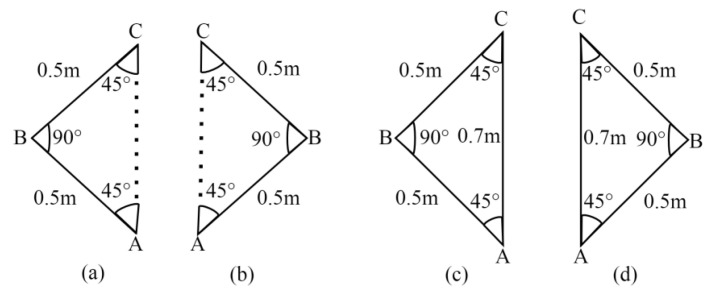
(**a**) ∆ABC for avoiding front, front and right obstacles. (**b**) ∆ABC for avoiding front and left obstacles. (**c**) Calculated straight distance after avoiding front, front and right obstacles. (**d**) Calculated straight distance (AC) after avoiding front and left obstacles.

Case 1:

If the obstacles are to the front, front and left, and front and right, then an isosceles right-angled triangle (∆ABC) is formed whose two sides AB and BC are known and the third side (AC) is unknown, as shown in Figure 3a,b.

Here, we used Pythagoras’ theorem to measure the correct distance. According to this theorem (Equation (1)), the sum of the square of the hypotenuse is equal to the sum of squares of the other two sides, i.e., base and perpendicular.

(Hypotenuse)^2^ = (Base)^2^ + (Perpendicular)^2^

(AC)^2^ = (AB)^2^ + (BC)^2^ (1)

In an isosceles right-angled triangle, both sides (AB and BC) have the same length (I).

So, (AC) ^2^ = (I)^2^ + (I)^2^

(AC) ^2^ = 2I^2^ (2)

$$\mathrm{AC}=\sqrt{2I^{2}}$$

After calculating the straight distance (Equation (2)) as shown in Figure 3c,d, the difference (*s*) between the 2I (base and perpendicular) and AC (hypotenuse) is measured to recalculate the path using Equation (3).

*s* = 2I − AC (3)

Finally, sis added with the traveled distance to attain the exact target.

**Figure 4:**
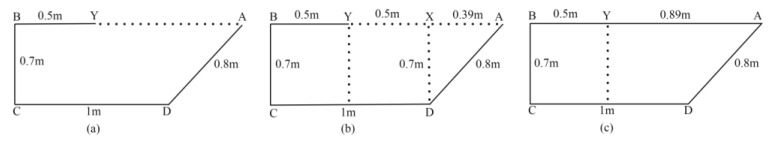
(**a**) Right trapezoid ABCD. (**b**) Right trapezoid with rectangle XBCD, and ∆AXD. (**c**) AY.

Case 2:

If the obstructions are to the front and left and right sides of the PT, then the OAM creates a right trapezoid (ABCD), as shown in Figure 4a. Where AB is the long base with a missing part AY, BC and DA are the legs, and CD is the short base, to find AY, we create a rectangle (XBCD) and a right triangle (AXD), as shown in Figure 4b. In the rectangle XBCD, XB = CD = 1 m, and BC = XD = 0.7 m. So, XY = XB − YB. Now, to find AX in the right triangle (AXD), we used Pythagoras’ theorem, i.e., (Hypotenuse)^2^ = (Base)^2^ + (Perpendicular)^2^. (DA)^2^ = (AX)^2^ + (XD)^2^ or (AX)^2^ = (DA)^2^ − (XD)^2^ or AX = $\sqrt{{\left(\mathrm{DA}\right)}^{2}-{\left(\mathrm{XD}\right)}^{2}}$. Now, AY = AX + XY, and YBCDA = YB + BC + CD + DA, as shown in Figure 4c. To recalculate the path, we find the difference (*f*) between YBCDA and AY. So, *f* = YBCDA−AY. Finally, *f* is added to the traveled distance to attain the exact target.

### 3.4. Navigation

The main aspect of designing a mobile robot is the navigational ability. The objective of navigation is to move from the starting point to the destination in a familiar or unfamiliar environment using the sensor values to perform a particular task. Mobile robots rely on various factors, including perception, localization, cognition, and control to achieve a specific goal. It becomes vital to provide the robot with useful information regarding its position to enable safe navigation.

The suggested framework functions in the indoor settings, GPS technology cannot be used for PT’s accurate location estimation. Consequently, we leveraged Perception-based positioning [48] which analyzes the distance, angle, and velocity to determine the robot’s current position. The location and direction of a mobile robotic device rely on identifying the target points. Autonomous robots collect information from encoders, odometers, infrared and ultrasonic sensors for position-based navigation. The robot’s location is confirmed by the sensor data, and position is determined by matching location parameters to a specific value [49].

### 3.5. Implementation

**Figure 5:**
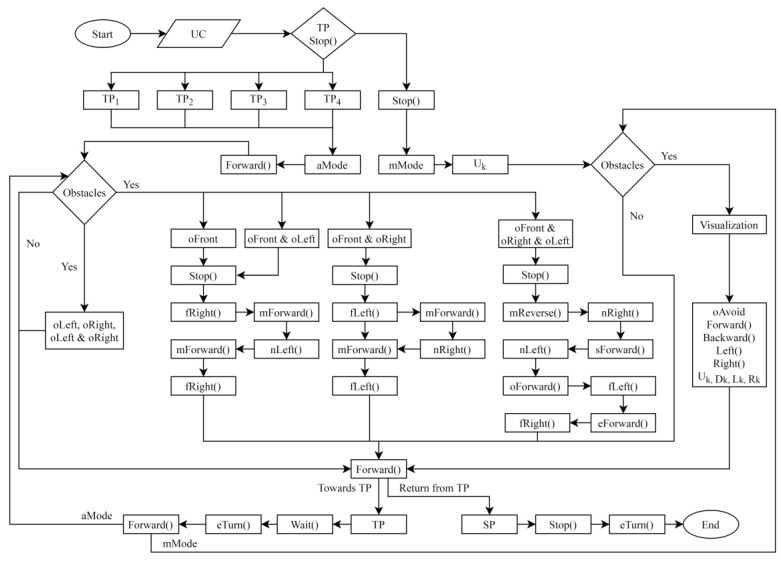
Flow chart for the decision-making algorithm.

The proposed navigation technique acquires different values from the sensors (LM393, MPU6050, and HC-SR04) which are checked by the decision-making algorithm, for navigation, as shown in Figure 5. The functions and keywords for the algorithm are shown in Table 1.

To perform remote monitoring of the patient, the system is initialized. After initialization, the PT waits for the user command.The operator issues the command by using the menu containing different buttons with labels, i.e., “Room 1” for target position 1 (TP_1_), “Room 2” for target position 2 (TP_2_), “Room 3” for target position 3 (TP_3_), “Room 4” for target position 4 (TP_4_), and “Stop” to halt PT’s motion and switch the driving mode to manual. When input parameters are received by the PT, they are evaluated by the algorithm. If the values are TP_1,_TP_2_, TP_3_, and TP_4_, the PT navigates in autonomous mode otherwise in manual mode. In autonomous mode, the PT begins to move forward towards the specific target based on the input value. During navigation, the ultrasonic sensors keep scanning for obstacles. If there are no obstructions, the PT moves straight towards the specific target position (TP). The accelerometer/gyroscope sensor (MPU6050) calculates the robot’s direction in the RE. The sensor values are utilized to align the PT on the straight path. If the rotation angle exceeds the predefined threshold (5°) in a particular direction, the PT is rotated in the opposite direction to align with the straight path. If there are barriers, the system uses the OAM to evade the obstacles, as described in Section 3.3.1. On reaching the destination, the PT stops moving and waits 10 s to collect and transmit health data. Then, it takes a turn (180°) and moves forward. The ultrasonic sensors continue scanning and if obstructions are detected, the OAM is executed to ensure safe navigation. On reaching the starting point, the PT stops, takes a turn (180°), and comes to a halt.

The health service can take control of the PT at any instant. When the operator issues the “Stop” command, the control is switched to the manual driving mode and the DTs stop moving. In manual mode, the user navigates the PT using the laptop’s arrow keys. If there are no obstructions, the PT moves straight to its target. If obstacles are detected by the PT, they are visualized in the VE. The operator observes the PT in the VE and avoids the barriers using the arrow keys. The “Up” arrow key is used to move forward; the “Down” arrow key is used to move backward; the “Left” key is used to turn left; and the “Right” key is used to turn right.

## 4. Experimental Setup and Performance Evaluation

**Figure 6:**
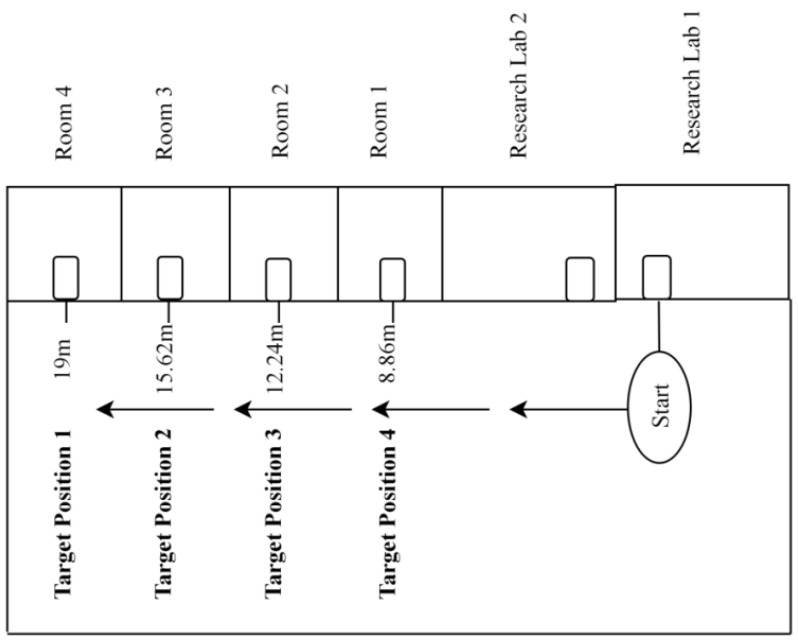
Experimental scenario of the proposed system.

The experimentation was carried out at the CS&IT department, University of Malakand. The aim was to move the PT to the desired destination autonomously by monitoring its VT in the VE and acquiring patient health information for transmission to the control station. The experimental environment is shown in Figure 6. Different target positions were specified to analyze the performance of the proposed system.

Target position 1(TP_1_):The health monitoring setup was attached to the human subject inside Room 4 (19 m from the starting point).

Target position 2 (TP_2_):The monitoring unit was mounted to the human subject inside Room 3 (15.62 m from the starting point).

Target position 3 (TP_3_):The patient monitoring setup was attached to the human subject inside Room 2 (12.24 m from the starting point).

Target position 4 (TP_4_):The health monitoring setup was attached to the human subject inside Room 1 (8.86 m from the starting point).

The proposed system’s performance was assessed by measuring navigation accuracy and monitoring data quality (DQ). Navigation accuracy was measured using the error, i.e., the difference between the actual distance and the distance covered by the PT. The health data wereevaluated by using the three well-known DQ dimensions (accuracy, completeness, and timeliness) [50]. The experimentation consisted of nine tasks (Task 1, Task 2, Task 3, Task 4, Task 5, Task 6, Task 7, Task 8, and Task 9). The tasks were classified into two categories. Category (Cat) 1, consisted of the tasks (1, 2, 3, and 4) classified according to the distance from the starting point. Category (Cat) 2 consisted of the tasks (5, 6, 7, 8, 9) classified based on various obstacles at different positions. Each task was performed 10 times, resulting 90 trials. The values for each task were averaged to obtainthe final result.

Cat 1:

Task 1: The PT had to reach TP_1_ and return to the control center after receiving the health data from the medical sensors.

Task 2: The PT had to reach TP_2_ and return to the control center after receiving health data from the medical sensors.

Task 3: The PT had to reach TP_3_ and return to the control center after receiving health data from the medical sensors.

Task 4: The PT had to reach TP_4_ and return to the control center after receiving health data from the medical sensors.

Cat 2:

Task 5: The PT had to reach TP_1_, avoiding a static obstacle (front) placed at TP_3_, and return to the control center after receiving health data from the medical sensors.

Task 6: The PT had to reach TP_1_, avoiding two static obstacles (front, left) placed at TP_3_, and return to the control center after receiving health data from the medical sensors.

Task 7: The PT had to reach TP_1_, avoiding two static obstacles (front, right) placed at TP_3_, and return to the control center after receiving health data from the medical sensors.

Task 8: The PT had to reach TP_1_, avoiding three static obstacles (front, right, left) placed at TP_3_, and return to the control center after receiving health data from the medical sensors.

Task 9: The PT had to reach TP_1_, avoiding a moving obstacle (front) initiated from TP_3_, and return to the control center after receiving sensor data from the medical sensors.

### 4.1. Navigation Accuracy

**Figure 7:**
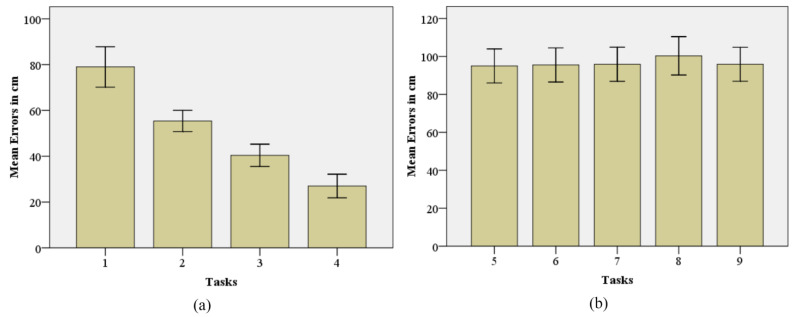
(**a**) ME and SD values of Cat 1; (**b**) ME and SD values of Cat 2.

The proposed system’s navigation accuracy is presented in Table 2 and Table 3. D_a_ is the actual distance in meters (m) from the starting point to the target position and then returning to the control station, and MD_c_ is the mean distance covered by PT during trials. The mean error (ME), and standard deviation (SD) of 10 trials for each task in Cat 1 are presented in Table 4, and the chart is shown in Figure 7a, whereas the ME, and SD of tasks in Cat 2 are presented in Table 5, and the chart is shown in Figure 7b.

To measure the statistical difference between the groups, we employed analysis of variance (ANOVA) [51].

The ANOVA test results in Table 6 show a significant variation *F*(3, 36) = 53.19, *p* = 0.000 among the Means of errors of Cat 1.

However, the ANOVA test results in Table 7 show no significant variation *F*(4, 45) = 0.22, *p* = 0.927 among the Means of errors of Cat 2.

### 4.2. Monitoring Data Quality

The monitoring data include heart rate, oxygen level, and temperature measurements. The oxygen level and heartbeat sensors have integer parameters. In contrast, temperature sensor measurements are gathered as float values in Celsius. When the PT reaches a specific target point, it connects with the PMU using the Bluetooth module to collect health parameters. The PMU includes various sensors: A heartbeat and oxygen sensor (MAX30100), and a temperature sensor (DS-18B20). It collects various information using the sensors attached to the human body (BS student with normal health status) inside the room. The DS-18B20 sensor is mounted to the wrist, while the MAX30100 sensor is attached to the subject’s forefinger. The sensors are connected to the microcontroller board (Arduino) that analyzes the collected information. A Bluetooth module is attached to the Arduino to transmit the analyzed data from the PMU to the PT. The PMU can be powered with a battery or electricity. The PT is also equipped with a Bluetooth module. When the PT arrives at the destination, a Bluetooth connection is established for data communication. After collecting sensor data from the PMU for 10 s, the PT sends it to the base station using the NRF24L01+ communication module. The collection and transmission of data by the PT occur in real time. The received parameters are then saved as an excel file to determine the DQ dimensions. The DQ dimensions are measured based on installing the monitoring setup at the maximum and minimum distances for data acquisition.

The accuracy and completeness of the monitoring data are computed by using Equations (4) and (5), respectively, as described in [52].

(4) $$\mathrm{Accuracy}\;=\;1-\frac{r_{e}}{r}$$

where *r_e_* is the number of erroneous data records and *r* is the total number of acquired data records.

(5) $$\mathrm{Completeness}\;=\;1-\frac{r_{c}}{r}$$

where *r_c_* is the number of not-null records and *r* is the total number of records received.

The timeliness of the health data is calculated using Equation (6) described in [53].

(6) $$\mathrm{Timeliness}\;=\;\frac{r_{o}}{r}$$

where *r_o_* is the number of data records acquired in a specified time interval and *r* is the total number of records in the same time slot.

Results of the DQ dimensions for Task 1, and Task 4 are shown in Table 8.

### 4.3. Comparative Analysis

The comparative analysis of the existing and the proposed systems in navigation is shown in Table 9. The accuracy of the proposed system is calculated by taking the mean of the tasks’(Task 1, 2, 3, 4, 5, 6, 7, 8, and 9) accuracies.

The comparative analysis of the DQ dimensions for the existing and proposed systems is shown in Table 10. The proposed system’s parameters are obtained by averaging the DQ dimensions of Tasks 1 and 4 of Table 8. The comparison of VR and DTs-based robotic systems is given in Table 11.

## 5. Discussion

The proposed approach creates a real-time RPM framework by implementing autonomous control of the PT in the DTs-based IoRT. The VE enables the operator to monitor the PT navigating autonomously towards the patient’s room for data collection. The designed algorithm can efficiently navigate the PT to the desired target location and return to the control center. The created OAM can detect and avoid obstacles in real time if they are present within a defined threshold distance, providing situational awareness to the operator by visualizing the obstacles, distance values, and distance labels (Left, Right, and Front) in the VE. It uses various geometrical patterns to recalculate the path and remove the distance errors. We evaluated the performance of OAM using the obstructions with predefined size (width = 20 cm, height = 35 cm). The barrier’s height has no impact on the system’s performance; however, the width value can affect the navigation accuracy. If the width of barriers is less than 20 cm, the PT can avoid them using the same functions. However, if the width exceeds 20 cm, the system should be re-programmed because it lacks dynamicity. Acquiring data from the monitoring unit for 10 s provides enough values to monitor the patient’s health. The navigation accuracy validates the PT’s efficiency. Task 4 in Cat 1, resulted in the highest accuracy values, i.e., 98.48 percent, while Task 1 resulted in the lowest accuracy score, i.e., 97.92 percent. Although the difference is less, the results show that short-distance tasks are more accurate than long-distance tasks. In Cat 2, Task 8 has relatively lower accuracy which means that increasing the number of barriers will slightly decrease the navigation accuracy. The ANOVA results indicate significant variation among the first experimental group (Cat 1). However, there is no significant variation among the second experimental group (Cat 2). In the evaluation of the DQ dimensions, it is found that the accuracy and completeness values are almost the same for both long and short-distance tasks (Task 1 and 4). However, the timeliness of data increases as distance increases.

The comparative analysis shows a clear advantage of the proposed system over the existing frameworks. In robot navigation, most systems lack evaluation protocols and navigation accuracy except the one developed in [33]. In RPM, only the framework proposed in [36] provides accuracy of monitoring data. However, it lacks the remaining two dimensions (completeness, and timeliness). The DTs and VR-based systems do not include a mechanism for connecting with external sensors or devices.

Unlike the existing systems, the proposed system provides a detailed evaluation protocol to calculate navigation accuracy and presents the monitoring DQ dimensions. Also, unlike the existing DTs and VR-based systems, the proposed framework can connect to external sensors and devices. Furthermore, our system is the sole one capable of monitoring the autonomous robotic device and remote patients.

The remaining part highlights the limitations of the proposed system to provide research directions for the new researchers. The proposed system uses a pre-built VE to visualize the PT’s virtual replica as well as its surroundings. However, if things change in real-time, e.g., objects added, removed, or displaced. It is very tricky to regularly update the virtual space according to the real scenario. The OAM’s performance has been examined for static-sized moving objects. However, no human subjects have been involved in experiments to assess the system’s performance at detecting and avoiding humans. Furthermore, the system does not include any mechanism to reflect the real scenario of patients.

## 6. Conclusions and Future Work

This research presented a real-time RPM system by implementing autonomous control inthe DTs-based IoRT. The pre-built virtual space presents the virtual replica of the PT and the RE. A Menu with several buttons is used to direct the PT to a specific location (patient room). A decision-making algorithm is developed that analyzes sensor data to enable safe navigation of the PT in the RE. The PT autonomously navigates to the patient room, collects and transmits health-related information, and returns to the health service. A real-time obstacle detection and avoidance mechanism is proposed that uses different geometrical patterns and mathematical formulae to evade obstructions and recalculate the path. The system allows the user to switch between the autonomous and manual driving modes. The experimental results and comparative analysis verify the proposed system’s advantage over the existing frameworks. The suggested system has an overall navigational accuracy of 97.81%, making it more effective compared to existing systems. In the context of DQ, the existing systems lack two dimensions: timeliness, and completeness. In data accuracy, our system has a clear advantage over the available frameworks showing an accuracy of 98.2%. In VR-based and DTs-based interfacing and supervision systems, the proposed system has a clear advantage over the other schemes as it includes autonomous control, which is missing in the majority of systems. Also, the proposed system provides connectivity with external sensors and detailed comparison, which the existing frameworks do not offer. In the future, the system will be upgraded to enable the PT to arrive at the patient room autonomously after a specific time interval rather than using the Menu for control. Machine learning techniques will be employed to predict the status of the patient and issue early warnings for prevention when anomalies in patient data are detected. Furthermore, the OAM will be improved to measure the obstacle’s size in real-time to enable dynamicity as it currently avoids barriers with predefined dimensions.

## Figures and Tables

**Table 1:** Functions and keywords for the algorithm.

S. No	Functions/Keywords	Description
1	Forward()	Moves forward
2	Backward()	Moves backward
3	Right()	Turns right
4	Left()	Turns left
5	nLeft()	Turns left 90°
6	fLeft()	Turns left 45°
7	fRight()	Turns right 45°
8	nRight()	Turns right 90°
9	eTurn()	Turns right 180°
10	mForward	Moves forward 0.5 m
11	sForward()	Moves forward 0.7 m
12	oForward()	Moves forward 1 m
13	eForward()	Moves forward 0.8 m
14	mReverse()	Moves backward 0.5
15	Stop()	Comes to halt
16	Wait()	Waits for 10 s
17	TP_1_	Target position 1 (19 m)
18	TP_2_	Target position 2 (15.62)
19	TP_3_	Target position 3 (12.24)
20	TP_4_	Target position 4 (8.86)
21	Dist	Distance
22	oLeft	Left obstacle distance less than 0.4 cm
23	oRight	Right obstacle distance less than 0.4 cm
24	oFront	Front obstacle distance less than 0.4 cm
25	U_k_	Upkey
26	D_k_	Down key
27	R_k_	Right key
28	L_k_	Left key
29	aMode	Autonomous mode
30	mMode	Manual mode
31	UC	User command
32	oAvoid	Obstacle avoidance
33	SP	Starting point

**Table 2:** Navigation accuracy obtained by averaging 10 trial values of each task in Cat 1.

Tasks	Target Positions	Obstacles	D_a_(m)	MD_c_ (m)	Error	Accuracy
Task 1	TP_1_	✕	38	37.21	2.08%	97.92%
Task 2	TP_2_	✕	31.24	30.69	1.76%	98.24%
Task 3	TP_3_	✕	24.48	24.08	1.63%	98.37%
Task 4	TP_4_	✕	17.72	17.45	1.52%	98.48%

**Table 3:** Navigation accuracy obtained by averaging 10 trial values of each task in Cat 2.

Tasks	Target Positions	Obstacles	Obstacles Status	Obstacles Positions	D_a_(m)	MD_c_ (m)	Error	Accuracy
Task 5	TP_1_	1	Static	TP_3_ (12.24 m) Front	38	37.05	2.50%	97.50%
Task 6	TP_1_	2	Static	TP_3_ Front, Left	38	37.05	2.50%	97.50%
Task 7	TP_1_	2	Static	TP_3_ Front, Right	38	37.04	2.53%	97.47%
Task 8	TP_1_	3	Static	TP_3_ Front, Right, Left	38	37	2.64%	97.37%
Task 9	TP_1_	1	Moving	TP_3_ Front	38	37.04	2.53%	97.47%

**Table 4:** ME and SD of 10 trial values for each task in Cat 1.

Tasks	ME (cm)	SD
Task 1	79	13.93
Task 2	55	7.31
Task 3	40	7.71
Task 4	27	8.18

**Table 5:** ME and SD of 10 trial values for each task in Cat 2.

Tasks	ME (cm)	SD
Task 5	95	14.18
Task 6	95.5	14.2
Task 7	95.9	14.19
Task 8	100.3	16.77
Task 9	95.9	14.14

**Table 6:** ANOVA test result for Cat 1.

	F	df	*p*-Value
Errors	53.19	3	0.012

**Table 7:** ANOVA test result for Cat 2.

	F	df	*p*-Value
Errors	0.22	4	0.985

**Table 8:** DQ dimensions of experimental Tasks 1 and 4 based on temperature, heartbeat and oxygen saturation sensors.

Tasks	Sensors	Accuracy	Completeness	Timeliness
Task 1	Heartbeat	0.977	0.984	0.967
	Oxygen	0.986	0.981	0.967
	Temperature	0.981	0.981	0.967
Task 4	Heartbeat	0.978	0.985	0.912
	Oxygen	0.987	0.986	0.912
	Temperature	0.984	0.982	0.912

**Table 9:** Comparative analysis of robot navigation systems.

Systems	Technologies	Connectivity	Interface	Mode	Services	Evaluation Protocol	Navigation Accuracy	Comparative Analysis
[31]	Robotics	Wi-Fi, Bluetooth	Desktop-based GUI	Manual	RPM	✕	✕	✕
[32]	Robotics	Bluetooth, Wi-Fi	Web-based	Autonomous	RPM, Detecting patients lying on floor	✕	✕	✕
[33]	Robotics	Wi-Fi	Web-based	Autonomous	Detecting infected patients, Surface disinfection	✓	85.5%	✕
[34]	IoRT	Wi-Fi, IEEE 802.22, Ethernet	Telegram bot interface	Autonomous	Elderly people monitoring	✕	✕	✕
[35]	Robotics, IoT	Wi-Fi	Mobile Application	Autonomous	RPM	✕	✕	✕
[36]	Robotics	ZigBee	GUI	Autonomous	RPM, Gait cycle assistance	✓	✕	✕
[37]	Robotics	Bluetooth, Internet	Android Application	Autonomous, Manual	RPM, Medicine delivery, Waste collection	✕	✕	✕
Proposed System	DTs-based IoRT	Bluetooth, NRF24L01+	Desktop-based VR	Autonomous, Manual	RPM	✓	97.81%	✓

**Table 10:** Comparative analysis of DQ dimensions calculated using temperature, heartbeat and oxygen sensors data.

Systems	Accuracy	Completeness	Timeliness	Comparative Analysis
[31]	✕	✕	✕	✕
[32]	✕	✕	✕	✕
[33]	✕	✕	✕	✕
[34]	✕	✕	✕	✕
[35]	✕	✕	✕	✕
[36]	0.970	✕	✕	✕
[37]	✕	✕	✕	✕
Proposed System	0.982	0.983	0.940	✓

**Table 11:** Comparative analysis of VR and DTs-based robotic systems.

Papers	Systems	Technologies	Description	Applications	Autonomous Operation	External Sensors Connectivity	Comparative Analysis
[38]	VR system	VR	Controlling mobile robot	Task inspection	✕	✕	✕
[39]	Robotic hand exoskeleton	VR, DT	Observing virtual objects	Rehabilitation	✕	✕	✕
[40]	WareVR	VR, DT	Monitoring autonomous robot	Transporting stock in a warehouse	✓	✕	✕
[41]	Robotic arm	VR, DT	Teleoperation	Conducting laboratory tests	✕	✕	✕
[42]	DTs-based robotic system	VR, DT	Controlling a FANUC robot	Industrial processes	✕	✕	✕
[43]	DTs-based robotic system	VR, DT	Controlling Industrial manufacturing robots	Industrial processes	✕	✕	✕
[44]	Robotic arm	VR, DT	To perform remote surgery	Medical purpose	✕	✕	✕
Proposed System	DTs-based IoRT	DTs, IoRT, VR	Control and monitor autonomous robot	RPM	✓	✓	✓

## Data Availability

The authors declare no concerns on data sharing.
